# Supplementary figures and images for: Tilapia lake virus causes mitochondrial damage: a proposed mechanism that leads to extensive death in fish cells
Source: PeerJ. 2023 Oct 5;11:e16190. doi: 10.7717/peerj.16190 (PMC10560495; doi:10.7717/peerj.16190)

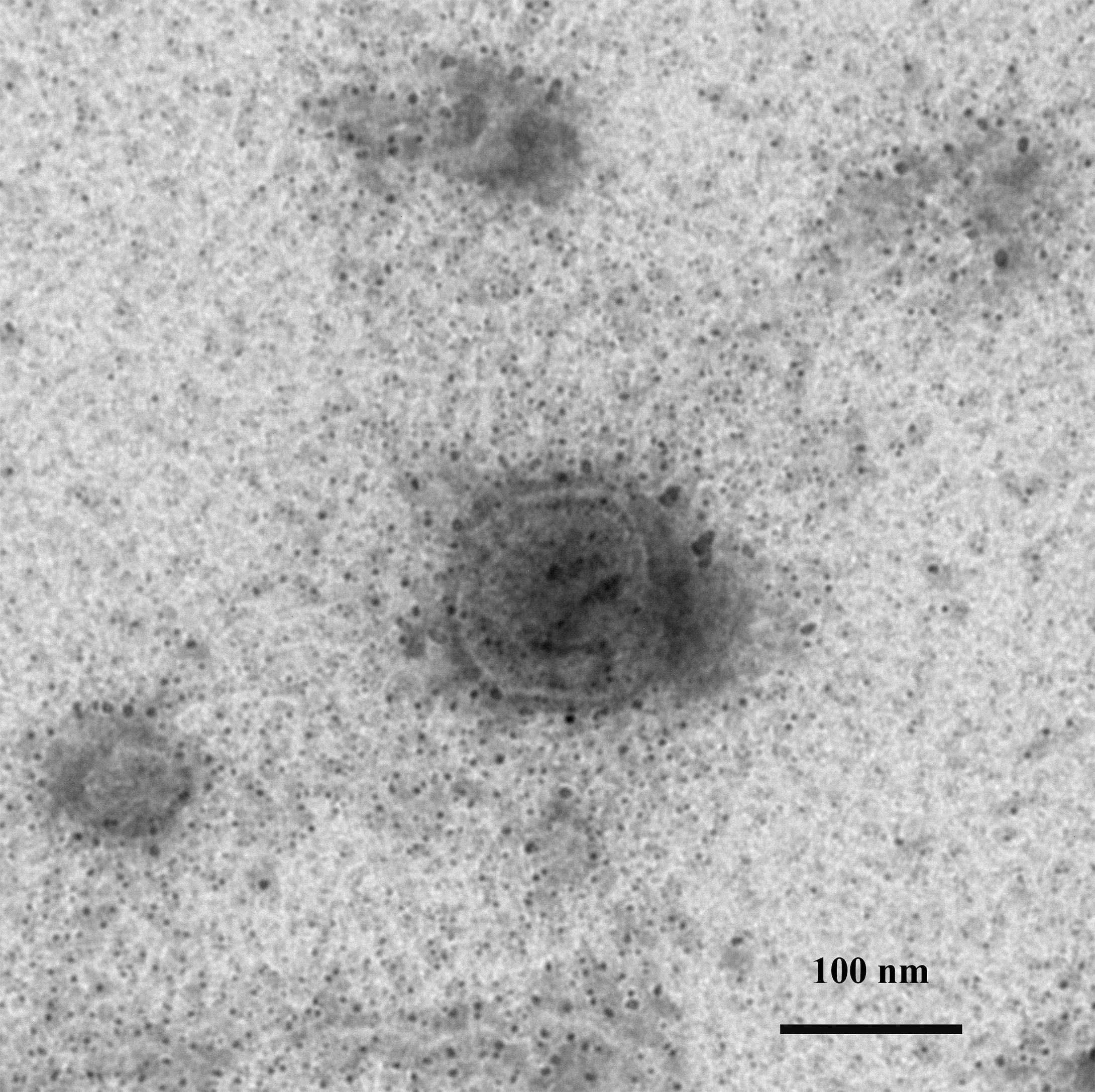

Supplement: Supplemental Information 1 — A round to oval shape at 50–120 nm with a central electron-dense core, surrounded by a capsid-like bilaminar structure. [file peerj-11-16190-s001.jpg]
